# Supplementary material for: Cervical Atrophy Following Complete Thoracic Spinal Cord Injury: Insights From a Multinational Cohort
Source: Eur J Neurol. 2026 Sep 10;33(9):e70750. doi: 10.1111/ene.70750 (PMC13560783; doi:10.1111/ene.70750)
Supplement: Supplementary file 1 [file ENE-33-e70750-s001.docx]

**Cervical atrophy following complete thoracic spinal cord injury: Insights from a multinational cohort**

**– Supplementary Material –**

Yann Quidé, PhD^1,2,*^, Negin Hesam-Shariati, PhD^1,2^, Zina Trost, PhD^3^, Thiago Folly de Campos, BPthy^1,2^, William R. Willoughby, PhD^4^, Mark Bolding, PhD^4^, Rachel E. Cowan, PhD^5^, Pauline Zahara, BPthy^1,2^, Sylvia M. Gustin, PhD^1,2^

^1^ NeuroRecovery Research Hub, School of Psychology, The University of New South Wales (UNSW) Sydney, Sydney, NSW, Australia

^2^ Centre for Pain IMPACT, Neuroscience Research Australia, Randwick, NSW, Australia

^3^ Department of Psychological and Brain Sciences, Texas A&M University, College Station, TX, USA

^4^ Department of Radiology, University of Alabama at Birmingham, Birmingham, AL, USA

^5^ Department of Physical Medicine & Rehabilitation, University of Alabama at Birmingham, Birmingham, AL, USA

*** Corresponding author**

Dr Yann Quidé, NeuroRecovery Research Hub, School of Psychology, Biological Sciences (Biolink) building, Level 1, UNSW Sydney, NSW, 2052, Australia.

| **Supplementary Table S1. Scanning parameters at all scanning sites** | | | |
| --- | --- | --- | --- |
| Scanning site | Scanner type | Sequence | Slice orientation |
| NeuRA1 | Philips Achieva TX 3T | 3D T1-weighted Magnetization Prepared Rapid Gradient Echo (MPRAGE) scans; TR/TE = 5.6 ms/2.5 ms, field of view = 250 × 250 × 174 mm, matrix 288 × 288, 200 slices, flip angle = 8°, voxel size 0.9 × 0.9 × 0.9 mm. 16 channels head coil | Sagittal |
| SVH | Philips Achieva TX 3T | 3D T1-weighted Magnetization Prepared Rapid Gradient Echo (MPRAGE) scans; TR/TE = 5.6 ms/2.5 ms, field of view = 250 × 250 × 174 mm, matrix 288 × 288, 200 slices, flip angle = 8°, voxel size 0.9 × 0.9 × 0.9 mm. 16 channels head coil | Sagittal |
| NeuRA2 | Philips Ingenia CX 3T | 3D T1-weighted Magnetization Prepared Rapid Gradient Echo (MPRAGE) scans; TR/TE = 8.0 ms/3.8 ms, field of view = 240 × 240 × 190 mm, matrix 268 × 268, 211 slices, flip angle = 8°, voxel size 0.9 × 0.9 × 0.9 mm. 32 channels head coil | Sagittal |
| HIRF | Siemens MAGNETOM Prisma 3T | 3D T1-weighted Magnetization Prepared Rapid Gradient Echo (MPRAGE) scans; TR/TE = 2400.0 ms/2.22 ms, field of view = 240 × 240 × 188 mm, matrix 288 × 288, 208 slices, flip angle = 8°, voxel size 0.8 × 0.8 × 0.9 mm. 64 channels head-neck coil | Sagittal |
| UAB | Siemens MAGNETOM Prisma 3T | 3D T1-weighted Magnetization Prepared Rapid Gradient Echo (MPRAGE) scans; TR/TE = 2400.0 ms/2.22 ms, field of view = 240 × 240 × 188 mm, matrix 288 × 288, 208 slices, flip angle = 8°, voxel size 0.8 × 0.8 × 0.9 mm. 64 channels head-neck coil | Sagittal |
| NeuRA: Neuroscience Research Australia, Randwick, NSW, Australia; SVH: St Vincent’s Hospital, Sydney, NSW, Australia; HIRF: Herston Imaging Research Facility, Herston, QLD, Australia; UAB: University of Alabama in Birmingham, Birmingham, AL, USA; TR: repetition time; TE: echo time | | | |

| **Supplementary Table S2. Effects of a thoracic SCI on c-spine morphometrics (C2 and C3 levels), when sites without controls (UAB, HIRF) were excluded** | | | | | | | | |
| --- | --- | --- | --- | --- | --- | --- | --- | --- |
| **C2 metric** | **Estimate** | **SE** | **95%LLCI** | **95%ULCI** | **t-value** | **p-value** | **pFDR** | **Cohen’s d** |
| **Mean CSA** | **-11.841** | **1.224** | **-14.240** | **-9.441** | **-9.673** | **1.163E-15** | **8.137E-15** | **-2.022** |
| **Mean AP** | **-0.847** | **0.102** | **-1.047** | **-0.647** | **-8.314** | **6.532E-13** | **2.286E-12** | **-1.706** |
| **Mean RL** | **-0.834** | **0.151** | **-1.129** | **-0.538** | **-5.527** | **2.846E-07** | **6.641E-07** | **-1.134** |
| **Eccentricity** | **0.026** | **0.010** | **0.006** | **0.047** | **2.547** | **0.012** | **0.022** | **0.527** |
| Solidity | -0.001 | 0.002 | -0.004 | 0.002 | -0.510 | 0.611 | 0.611 | -0.105 |
| Orientation | 0.427 | 0.367 | -0.292 | 1.146 | 1.164 | 0.247 | 0.346 | 0.239 |
| Length | 0.184 | 0.335 | -0.472 | 0.840 | 0.550 | 0.584 | 0.611 | 0.113 |
| **C3 metric** | **Estimate** | **SE** | **LLCI** | **ULCI** | **t-value** | **p-value** | **pFDR** | **Cohen’s d** |
| **Mean CSA** | **-10.881** | **1.324** | **-13.476** | **-8.285** | **-8.216** | **1.053E-12** | **7.372E-12** | **-1.686** |
| **Mean AP** | **-0.830** | **0.108** | **-1.041** | **-0.619** | **-7.712** | **1.210E-11** | **4.235E-11** | **-1.583** |
| **Mean RL** | **-0.750** | **0.160** | **-1.063** | **-0.436** | **-4.684** | **9.381E-06** | **2.189E-05** | **-0.962** |
| **Eccentricity** | **0.027** | **0.009** | **0.010** | **0.044** | **3.098** | **0.003** | **0.004** | **0.636** |
| Solidity | -0.002 | 0.002 | -0.005 | 0.001 | -1.205 | 0.231 | 0.324 | -0.247 |
| Orientation | 0.292 | 0.358 | -0.409 | 0.994 | 0.817 | 0.416 | 0.416 | 0.168 |
| Length | 0.368 | 0.407 | -0.429 | 1.166 | 0.906 | 0.368 | 0.416 | 0.187 |
| SCI: spinal cord injury; SE: standard error; LLCI/ULCI: lower-level and upper-level 95% confidence interval; FDR: false discovery rate correction; CSA: cross-sectional area; AP: anteroposterior diameter; RL: right-left diameter  Group differences surviving FDR correction are in bold | | | | | | | | |

| **Supplementary Table S3. Effects of a thoracic SCI on c-spine morphometrics (C2 and C3 levels), among people who experienced or not chronic neuropathic pain, when sites without controls (UAB, HIRF) were excluded** | | | | | | | | | | | | | | | | |  |
| --- | --- | --- | --- | --- | --- | --- | --- | --- | --- | --- | --- | --- | --- | --- | --- | --- | --- |
| **C2 metric** | | **Estimate** | **SE** | | **95%LLCI** | | **95%ULCI** | | **t-value** | | **p-value** | | **pFDR** | | **Cohen’s d** | |  |
| ***SCI-NP vs HC*** |  | | |  | |  | |  | |  | |  | |  | |  | |
| **Mean CSA** | | **-10.398** | **1.455** | | **-13.249** | | **-7.547** | | **-7.149** | | **2.160E-10** | | **1.512E-09** | | **-1.501** | |  |
| **Mean AP** | | **-0.801** | **0.122** | | **-1.040** | | **-0.562** | | **-6.564** | | **3.084E-09** | | **1.145E-08** | | **-1.372** | |  |
| **Mean RL** | | **-0.687** | **0.173** | | **-1.027** | | **-0.347** | | **-3.965** | | **1.428E-04** | | **3.334E-04** | | **-0.818** | |  |
| **Eccentricity** | | **0.032** | **0.012** | | **0.009** | | **0.056** | | **2.687** | | **0.009** | | **0.018** | | **0.609** | |  |
| Solidity | | -0.001 | 0.002 | | -0.004 | | 0.003 | | -0.504 | | 0.615 | | 0.718 | | -0.104 | |  |
| Orientation | | 0.453 | 0.428 | | -0.385 | | 1.291 | | 1.059 | | 0.292 | | 0.455 | | 0.218 | |  |
| Length | | 0.254 | 0.395 | | -0.521 | | 1.029 | | 0.642 | | 0.523 | | 0.666 | | 0.141 | |  |
| ***SCI-nonNP vs HC*** | |  |  | |  | |  | |  | |  | |  | |  | |  |
| **Mean CSA** | | **-13.433** | **1.644** | | **-16.655** | | **-10.211** | | **-8.171** | | **1.528E-12** | | **2.139E-11** | | **-1.698** | |  |
| **Mean AP** | | **-0.901** | **0.138** | | **-1.171** | | **-0.631** | | **-6.544** | | **3.270E-09** | | **1.145E-08** | | **-1.360** | |  |
| **Mean RL** | | **-1.068** | **0.205** | | **-1.470** | | **-0.667** | | **-5.216** | | **1.086E-06** | | **3.041E-06** | | **-1.076** | |  |
| Eccentricity | | 0.018 | 0.014 | | -0.009 | | 0.046 | | 1.297 | | 0.198 | | 0.346 | | 0.268 | |  |
| Solidity | | -0.001 | 0.002 | | -0.005 | | 0.004 | | -0.281 | | 0.780 | | 0.839 | | -0.058 | |  |
| Orientation | | 0.386 | 0.505 | | -0.605 | | 1.376 | | 0.763 | | 0.447 | | 0.626 | | 0.157 | |  |
| Length | | 0.093 | 0.456 | | -0.802 | | 0.988 | | 0.204 | | 0.839 | | 0.839 | | 0.042 | |  |
| ***SCI-NP vs SCI-nonNP*** | | | |  | |  | |  | |  | |  | |  | |  | |
| Mean CSA | | 3.035 | 1.895 | | -0.679 | | 6.749 | | 1.601 | | 0.113 | | 0.316 | | 0.335 | |  |
| Mean AP | | 0.100 | 0.159 | | -0.211 | | 0.412 | | 0.630 | | 0.530 | | 0.825 | | 0.131 | |  |
| Mean RL | | 0.381 | 0.228 | | -0.065 | | 0.827 | | 1.674 | | 0.097 | | 0.316 | | 0.345 | |  |
| Eccentricity | | 0.014 | 0.016 | | -0.017 | | 0.045 | | 0.899 | | 0.371 | | 0.742 | | 0.196 | |  |
| Solidity | | -3.140E-04 | 0.002 | | -0.005 | | 0.004 | | -0.131 | | 0.896 | | 0.905 | | -0.027 | |  |
| Orientation | | 0.067 | 0.562 | | -1.034 | | 1.168 | | 0.119 | | 0.905 | | 0.905 | | 0.025 | |  |
| Length | | 0.161 | 0.516 | | -0.852 | | 1.173 | | 0.311 | | 0.756 | | 0.905 | | 0.067 | |  |
|  | |  |  | |  | |  | |  | |  | |  | |  | |  |
| **C3 metric** | | **Estimate** | **SE** | | **95%LLCI** | | **95%ULCI** | | **t-value** | | **p-value** | | **pFDR** | | **Cohen’s d** | |  |
| ***SCI-NP vs HC*** | |  |  | |  | |  | |  | |  | |  | |  | |  |
| **Mean CSA** | | **-9.757** | **1.579** | | **-12.852** | | **-6.662** | | **-6.179** | | **1.729E-08** | | **1.210E-07** | | **-1.287** | |  |
| **Mean AP** | | **-0.797** | **0.127** | | **-1.046** | | **-0.548** | | **-6.272** | | **2.323E-07** | | **8.131E-07** | | **-2.026** | |  |
| **Mean RL** | | **-0.617** | **0.191** | | **-0.990** | | **-0.243** | | **-3.236** | | **0.002** | | **0.004** | | **-0.680** | |  |
| Eccentricity | | **0.028** | **0.010** | | **0.008** | | **0.049** | | **2.790** | | **0.006** | | **0.013** | | **0.575** | |  |
| Solidity | | -0.002 | 0.002 | | -0.006 | | 0.002 | | -1.041 | | 0.301 | | 0.421 | | -0.222 | |  |
| Orientation | | 0.281 | 0.417 | | -0.536 | | 1.098 | | 0.674 | | 0.502 | | 0.572 | | 0.139 | |  |
| Length | | 0.594 | 0.479 | | -0.345 | | 1.533 | | 1.241 | | 0.218 | | 0.340 | | 0.273 | |  |
| ***SCI-nonNP vs HC*** |  | | |  | |  | |  | |  | |  | |  | |  | |
| **Mean CSA** | | **-12.251** | **1.779** | | **-15.739** | | **-8.764** | | **-6.885** | | **6.815E-10** | | **9.542E-09** | | **-1.432** | |  |
| **Mean AP** | | **-0.858** | **0.148** | | **-1.148** | | **-0.568** | | **-5.790** | | **9.321E-08** | | **4.350E-07** | | **-1.197** | |  |
| **Mean RL** | | **-0.917** | **0.215** | | **-1.340** | | **-0.495** | | **-4.258** | | **4.942E-05** | | **1.384E-04** | | **-0.885** | |  |
| Eccentricity | | 0.025 | 0.012 | | 0.001 | | 0.049 | | 2.073 | | 0.041 | | 0.072 | | 0.428 | |  |
| Solidity | | -0.002 | 0.002 | | -0.006 | | 0.002 | | -0.859 | | 0.392 | | 0.499 | | -0.178 | |  |
| Orientation | | 0.310 | 0.493 | | -0.656 | | 1.277 | | 0.629 | | 0.531 | | 0.572 | | 0.130 | |  |
| Length | | 0.058 | 0.554 | | -1.027 | | 1.143 | | 0.105 | | 0.917 | | 0.917 | | 0.022 | |  |
| ***SCI-NP vs SCI-nonNP*** | | | |  | |  | |  | |  | |  | |  | |  | |
| Mean CSA | | 2.494 | 2.057 | | -1.538 | | 6.526 | | 1.212 | | 0.228 | | 0.534 | | 0.252 | |  |
| Mean AP | | 0.061 | 0.166 | | -0.265 | | 0.387 | | 0.365 | | 0.717 | | 0.961 | | 0.101 | |  |
| Mean RL | | 0.301 | 0.248 | | -0.186 | | 0.787 | | 1.212 | | 0.229 | | 0.534 | | 0.254 | |  |
| Eccentricity | | 0.003 | 0.013 | | -0.023 | | 0.030 | | 0.258 | | 0.797 | | 0.961 | | 0.053 | |  |
| Solidity | | -1.224E-04 | 0.002 | | -0.005 | | 0.005 | | -0.049 | | 0.961 | | 0.961 | | -0.010 | |  |
| Orientation | | -0.029 | 0.548 | | -1.103 | | 1.045 | | -0.053 | | 0.958 | | 0.961 | | -0.011 | |  |
| Length | | 0.536 | 0.626 | | -0.690 | | 1.763 | | 0.857 | | 0.394 | | 0.689 | | 0.185 | |  |
| SCI: spinal cord injury; SCI-NP: people with SCI and chronic neuropathic pain; SCI-nonNP: people with SCI and without chronic neuropathic pain; SE: standard error; LLCI/ULCI: lower-level and upper-level 95% confidence interval; FDR: false discovery rate correction; CSA: cross-sectional area; AP: anteroposterior diameter; RL: right-left diameter  Group differences surviving FDR correction are in bold | | | | | | | | | | | | | | | | |  |

| **Supplementary Table S4. Associations between c-spine morphometrics (C2 and C3 levels) and age for the control, the combined SCI, the SCI-NP and SCI-nonNP groups** | | | | | |
| --- | --- | --- | --- | --- | --- |
| **Controls** |  |  |  |  |  |
| **C2 metric** | **Estimate** | **SE** | **t-value** | **p-value** | **pFDR** |
| **Mean CSA** | **-0.156** | **0.057** | **-2.758** | **0.008** | **0.033** |
| **Mean AP** | **-0.013** | **0.005** | **-2.689** | **0.009** | **0.033** |
| Mean RL | -0.008 | 0.006 | -1.339 | 0.186 | 0.260 |
| Eccentricity | 6.637E-4 | 4.912E-4 | 1.351 | 0.182 | 0.260 |
| Solidity | 5.727E-5 | 7.451E-5 | 0.769 | 0.446 | 0.446 |
| Orientation | -0.026 | 0.016 | -1.609 | 0.113 | 0.260 |
| Length | -0.014 | 0.016 | -0.901 | 0.372 | 0.434 |
| **C3 metric** |  |  |  |  |  |
| **Mean CSA** | **-0.180** | **0.059** | **-3.035** | **0.004** | **0.025** |
| **Mean AP** | **-0.015** | **0.005** | **-2.790** | **0.007** | **0.025** |
| Mean RL | -0.009 | 0.006 | -1.429 | 0.159 | 0.319 |
| Eccentricity | 0.001 | 4.422E-04 | 1.350 | 0.182 | 0.319 |
| Solidity | -6.034E-05 | 7.617E-05 | -0.792 | 0.432 | 0.504 |
| Orientation | 0.003 | 0.015 | 0.194 | 0.847 | 0.847 |
| Length | -0.020 | 0.019 | -1.027 | 0.309 | 0.432 |
| **SCI (all)** |  |  |  |  |  |
| **C2 metric** | **Estimate** | **SE** | **t-value** | **p-value** | **pFDR** |
| Mean CSA | -0.082 | 0.063 | -1.306 | 0.197 | 0.393 |
| Mean AP | -0.003 | 0.004 | -0.708 | 0.482 | 0.563 |
| Mean RL | -0.011 | 0.009 | -1.229 | 0.224 | 0.393 |
| Eccentricity | - 1.984E-04 | 4.230E-4 | -0.469 | 0.6418 | 0.641 |
| Solidity | - 1.573E-04 | 6.612E-5 | -2.379 | 0.021 | 0.231 |
| Orientation | 0.026 | 0.019 | 1.366 | 0.178 | 0.383 |
| Length | -0.015 | 0.018 | -0.870 | 0.389 | 0.544 |
| **C3 metric** |  |  |  |  |  |
| Mean CSA | -0.112 | 0.069 | -1.620 | 0.112 | 0.376 |
| Mean AP | -0.006 | 0.005 | -1.128 | 0.265 | 0.376 |
| Mean RL | -0.012 | 0.009 | -1.335 | 0.188 | 0.376 |
| Eccentricity | 1.044E-05 | 3.174E-04 | 0.033 | 0.974 | 0.974 |
| Solidity | 1.392E-04 | 6.898E-05 | -2.018 | 0.049 | 0.346 |
| Orientation | 0.026 | 0.023 | 1.109 | 0.272 | 0.462 |
| Length | -0.023 | 0.021 | -1.120 | 0.268 | 0.376 |
| **SCI-NP** |  |  |  |  |  |
| **C2 metric** | **Estimate** | **SE** | **t-value** | **p-value** | **pFDR** |
| Mean CSA | -0.079 | 0.082 | -0.964 | 0.343 | 0.541 |
| Mean AP | -0.002 | 0.005 | -0.380 | 0.706 | 0.707 |
| Mean RL | -0.012 | 0.012 | -0.972 | 0.338 | 0.541 |
| Eccentricity | - 2.423E-04 | 5.436E-4 | -0.446 | 0.659 | 0.707 |
| **Solidity** | **-2.530E-4** | **8.209E-5** | **-3.082** | **0.004** | **0.028** |
| Orientation | 0.046 | 0.023 | 2.020 | 0.051 | 0.179 |
| Length | -0.018 | 0.020 | -0.879 | 0.387 | 0.541 |
| **C3 metric** |  |  |  |  |  |
| Mean CSA | -0.095 | 0.093 | -1.020 | 0.316 | 0.656 |
| Mean AP | -0.004 | 0.007 | -0.586 | 0.563 | 0.656 |
| Mean RL | -0.011 | 0.013 | -0.879 | 0.385 | 0.656 |
| Eccentricity | -4.589E-05 | 3.925E-04 | -0.117 | 0.908 | 0.908 |
| Solidity | -2.164E-04 | 8.241E-05 | -2.626 | 0.013 | 0.094 |
| Orientation | 0.016 | 0.027 | 0.585 | 0.563 | 0.656 |
| Length | -0.029 | 0.022 | -1.340 | 0.190 | 0.656 |
| **SCI-nonNP** | |  |  |  |  |
| **C2 metric** | **Estimate** | **SE** | **t-value** | **p-value** | **pFDR** |
| Mean CSA | -0.136 | 0.081 | -1.680 | 0.111 | 0.389 |
| Mean AP | -0.006 | 0.007 | -0.911 | 0.375 | 0.601 |
| Mean RL | -0.017 | 0.008 | -1.992 | 0.063 | 0.389 |
| Eccentricity | -4.119E-04 | 6.194E-4 | -0.665 | 0.515 | 0.601 |
| Solidity | 8.404E-5 | 9.372E-5 | 0.897 | 0.382 | 0.601 |
| Orientation | -0.025 | 0.0334 | -0.744 | 0.467 | 0.601 |
| Length | -0.012 | 0.035 | -0.339 | 0.739 | 0.739 |
| **C3 metric** |  |  |  |  |  |
| Mean CSA | -0.168 | 0.077 | -2.190 | 0.043 | 0.277 |
| Mean AP | -0.011 | 0.007 | -1.629 | 0.122 | 0.284 |
| Mean RL | -0.017 | 0.009 | -1.867 | 0.079 | 0.277 |
| Eccentricity | 1.728E-05 | 0.001 | 0.031 | 0.975 | 0.975 |
| Solidity | 7.952E-04 | 1.186E-04 | 0.670 | 0.512 | 0.895 |
| Orientation | 0.005 | 0.032 | 0.163 | 0.873 | 0.975 |
| Length | -0.014 | 0.044 | -0.324 | 0.750 | 0.975 |
| SCI: spinal cord injury; SE: standard error; LLCI/ULCI: lower-level and upper-level 95% confidence interval; FDR: false discovery rate correction; CSA: cross-sectional area; AP: anteroposterior diameter; RL: right-left diameter  Associations surviving FDR correction are in bold | | | | | |

| **Supplementary Table S5. Associations between c-spine morphometrics (C2 and C3 levels) and time since injury for the combined SCI, the SCI-NP and SCI-nonNP groups** | | | | | |
| --- | --- | --- | --- | --- | --- |
| **SCI (all)** |  |  |  |  |  |
| **C2 metric** | **Estimate** | **SE** | **t-value** | **p-value** | **pFDR** |
| Mean CSA | -0.114 | 0.069 | -1.643 | 0.106 | 0.248 |
| Mean AP | -0.002 | 0.005 | -0.428 | 0.670 | 0.782 |
| Mean RL | -0.021 | 0.009 | -2.284 | 0.027 | 0.133 |
| Eccentricity | -0.001 | 4.534E-4 | -2.129 | 0.038 | 0.133 |
| Solidity | 1.596E-5 | 7.767E-5 | 0.205 | 0.838 | 0.838 |
| Orientation | 0.030 | 0.021 | 1.427 | 0.160 | 0.279 |
| Length | 0.024 | 0.020 | 1.186 | 0.241 | 0.338 |
| **C3 metric** |  |  |  |  |  |
| Mean CSA | -0.102 | 0.078 | -1.307 | 0.197 | 0.462 |
| Mean AP | -0.004 | 0.006 | -0.639 | 0.526 | 0.526 |
| Mean RL | -0.010 | 0.010 | -1.012 | 0.316 | 0.462 |
| Eccentricity | 3.292E-04 | 3.511E-04 | -0.938 | 0.353 | 0.462 |
| Solidity | 6.957E-05 | 8126E-05 | -0.856 | 0.396 | 0.462 |
| Orientation | 0.026 | 0.023 | 1.109 | 0.272 | 0.462 |
| Length | 0.021 | 0.023 | 0.917 | 0.363 | 0.462 |
| **SCI-NP** |  |  |  |  |  |
| **C2 metric** | **Estimate** | **SE** | **t-value** | **p-value** | **pFDR** |
| Mean CSA | -0.079 | 0.110 | -0.720 | 0.477 | 0.668 |
| Mean AP | 0.001 | 0.007 | 0.175 | 0.862 | 0.862 |
| Mean RL | -0.019 | 0.016 | -1.241 | 0.223 | 0.390 |
| Eccentricity | -0.001 | 7.005E-4 | -1.602 | 0.118 | 0.287 |
| Solidity | - 6.069E-05 | 1.228E-4 | -0.494 | 0.624 | 0.728 |
| Orientation | 0.049 | 0.031 | 1.581 | 0.123 | 0.287 |
| Length | 0.049 | 0.027 | 1.844 | 0.075 | 0.287 |
| **C3 metric** |  |  |  |  |  |
| Mean CSA | -0.075 | 0.125 | -0.597 | 0.555 | 0.777 |
| Mean AP | -3.225E-04 | 0.009 | -0.036 | 0.971 | 0.971 |
| Mean RL | -0.011 | 0.017 | -0.642 | 0.525 | 0.777 |
| Eccentricity | -0.001 | 0.001 | -1.087 | 0.285 | 0.664 |
| Solidity | -1.912E-04 | 1.171E-04 | -1.633 | 0.113 | 0.394 |
| Orientation | 0.008 | 0.036 | 0.218 | 0.829 | 0.967 |
| Length | 0.049 | 0.028 | 1.706 | 0.098 | 0.394 |
| **SCI-nonNP** |  |  |  |  |  |
| **C2 metric** | **Estimate** | **SE** | **t-value** | **p-value** | **pFDR** |
| Mean CSA | -0.140 | 0.070 | -2.010 | 0.061 | 0.212 |
| Mean AP | -0.006 | 0.006 | -0.931 | 0.365 | 0.511 |
| Mean RL | -0.019 | 0.007 | -2.826 | 0.012 | 0.082 |
| Eccentricity | -0.001 | 0.001 | -1.125 | 0.276 | 0.483 |
| Solidity | 9.817E-05 | 8.161E-05 | 1.203 | 0.246 | 0.483 |
| Orientation | 0.017 | 0.030 | 0.582 | 0.568 | 0.663 |
| Length | -0.010 | 0.031 | -0.321 | 0.752 | 0.752 |
| **C3 metric** |  |  |  |  |  |
| Mean CSA | -0.106 | 0.072 | -1.461 | 0.162 | 0.449 |
| Mean AP | -0.007 | 0.006 | -1.174 | 0.257 | 0.449 |
| Mean RL | -0.011 | 0.008 | -1.257 | 0.226 | 0.449 |
| Eccentricity | 3.389E-05 | 4.899E-04 | 0.069 | 0.946 | 0.946 |
| Solidity | 6.838E-05 | 1.052E-05 | 0.650 | 0.525 | 0.734 |
| Orientation | 0.055 | 0.025 | 2.188 | 0.043 | 0.300 |
| Length | -0.012 | 0.039 | -0.299 | 0.769 | 0.897 |
| SCI: spinal cord injury; SE: standard error; FDR: false discovery rate correction; CSA: cross-sectional area; AP: anteroposterior diameter; RL: right-left diameter  Associations surviving FDR correction are in bold | | | | | |

| **Supplementary Table S6. Associations between c-spine morphometrics (C2 and C3 levels) and levels of injury (when considered at upper, mean or lower levels when lesions spread over multiple levels) for the combined SCI, the SCI-NP and SCI-nonNP groups** | | | | | |
| --- | --- | --- | --- | --- | --- |
| **SCI (all)** |  |  |  |  |  |
| **C2 metric** | **Estimate** | **SE** | **t-value** | **p-value** | **pFDR** |
| ***Upper*** |  |  |  |  |  |
| **Mean CSA** | **0.587** | **0.115** | **5.091** | **5.212E-06** | **3.649E-05** |
| **Mean AP** | **0.355** | **0.131** | **2.715** | **9.208E-03** | **0.022** |
| **Mean RL** | **0.568** | **0.117** | **4.854** | **1.184E-05** | **4.144E-05** |
| Eccentricity | 0.191 | 0.139 | 1.372 | 0.176 | 0.308 |
| Solidity | -0.071 | 0.141 | -0.502 | 0.618 | 0.679 |
| Orientation | -0.129 | 0.140 | -0.919 | 0.363 | 0.508 |
| Length | -0.057 | 0.136 | -0.417 | 0.679 | 0.679 |
| ***Mean*** |  |  |  |  |  |
| **Mean CSA** | **0.574** | **0.116** | **4.969** | **7.976E-06** | **5.583E-05** |
| **Mean AP** | **0.351** | **0.130** | **2.709** | **9.366E-03** | **0.022** |
| **Mean RL** | **0.558** | **0.117** | **4.766** | **1.602E-05** | **5.607E-05** |
| Eccentricity | 0.191 | 0.138 | 1.385 | 0.172 | 0.301 |
| Solidity | -0.070 | 0.140 | -0.499 | 0.620 | 0.620 |
| Orientation | -0.133 | 0.140 | -0.953 | 0.345 | 0.483 |
| Length | -0.090 | 0.135 | -0.671 | 0.506 | 0.590 |
| ***Lower*** |  |  |  |  |  |
| **Mean CSA** | **0.574** | **0.116** | **4.939** | **8.839E-06** | **5.800E-05** |
| **Mean AP** | **0.350** | **0.130** | **2.698** | **0.010** | **0.022** |
| **Mean RL** | **0.559** | **0.118** | **4.757** | **1.657E-05** | **5.800E-05** |
| Eccentricity | 0.196 | 0.139 | 1.412 | 0.164 | 0.287 |
| Solidity | -0.079 | 0.141 | -0.564 | 0.576 | 0.576 |
| Orientation | -0.135 | 0.140 | -0.966 | 0.339 | 0.461 |
| Length | -0.116 | 0.135 | -0.858 | 0.395 | 0.461 |
| **C3 metric** |  |  |  |  |  |
| ***Upper*** |  |  |  |  |  |
| **Mean CSA** | **0.551** | **0.119** | **4.637** | **2.49E-05** | **8.73E-05** |
| Mean AP | 0.296 | 0.135 | 2.185 | 0.034 | 0.059 |
| **Mean RL** | **0.603** | **0.114** | **5.310** | **2.41E-06** | **1.69E-05** |
| **Eccentricity** | **0.358** | **0.132** | **2.701** | **0.009** | **0.022** |
| Solidity | -0.185 | 0.137 | -1.345 | 0.185 | 0.259 |
| Orientation | 0.063 | 0.141 | 0.444 | 0.659 | 0.768 |
| Length | -0.012 | 0.139 | -0.084 | 0.933 | 0.933 |
| ***Mean*** |  |  |  |  |  |
| **Mean CSA** | **0.528** | **0.120** | **4.407** | **5.40E-05** | **1.89E-04** |
| Mean AP | 0.289 | 0.135 | 2.139 | 0.037 | 0.065 |
| **Mean RL** | **0.584** | **0.115** | **5.089** | **5.25E-06** | **3.68E-05** |
| **Eccentricity** | **0.338** | **0.133** | **2.549** | **0.014** | **0.032** |
| Solidity | -0.183 | 0.136 | -1.340 | 0.187 | 0.261 |
| Orientation | 0.057 | 0.141 | 0.404 | 0.688 | 0.712 |
| Length | -0.051 | 0.137 | -0.371 | 0.712 | 0.712 |
| ***Lower*** |  |  |  |  |  |
| **Mean CSA** | **0.523** | **0.121** | **4.334** | **6.89E-05** | **2.41E-04** |
| Mean AP | 0.290 | 0.135 | 2.148 | 0.037 | 0.064 |
| **Mean RL** | **0.579** | **0.116** | **5.014** | **6.81E-06** | **4.77E-05** |
| **Eccentricity** | **0.325** | **0.134** | **2.435** | **0.018** | **0.043** |
| Solidity | -0.190 | 0.136 | -1.395 | 0.170 | 0.237 |
| Orientation | 0.054 | 0.141 | 0.381 | 0.704 | 0.704 |
| Length | -0.081 | 0.138 | -0.592 | 0.556 | 0.649 |
| **SCI-NP** |  |  |  |  |  |
| **C2 metric** | **Estimate** | **SE** | **t-value** | **p-value** | **pFDR** |
| ***Upper*** |  |  |  |  |  |
| **Mean CSA** | **0.660** | **0.132** | **4.998** | **1.726E-05** | **1.208E-04** |
| **Mean AP** | **0.394** | **0.156** | **2.527** | **0.017** | **0.039** |
| **Mean RL** | **0.607** | **0.139** | **4.359** | **1.144E-04** | **4.003E-04** |
| Eccentricity | 0.243 | 0.169 | 1.442 | 0.158 | 0.270 |
| Solidity | -0.168 | 0.171 | -0.978 | 0.335 | 0.391 |
| Orientation | -0.225 | 0.170 | -1.330 | 0.193 | 0.270 |
| Length | -0.134 | 0.165 | -0.814 | 0.422 | 0.422 |
| ***Mean*** |  |  |  |  |  |
| **Mean CSA** | **0.645** | **0.133** | **4.839** | **2.779E-05** | **1.945E-04** |
| **Mean AP** | **0.398** | **0.154** | **2.584** | **0.015** | **0.034** |
| **Mean RL** | **0.594** | **0.140** | **4.242** | **1.609E-04** | **5.632E-04** |
| Eccentricity | 0.241 | 0.168 | 1.436 | 0.160 | 0.280 |
| Solidity | -0.154 | 0.171 | -0.899 | 0.375 | 0.375 |
| Orientation | -0.220 | 0.169 | -1.301 | 0.202 | 0.283 |
| Length | -0.147 | 0.163 | -0.903 | 0.374 | 0.375 |
| ***Lower*** |  |  |  |  |  |
| **Mean CSA** | **0.630** | **0.136** | **4.642** | **4.977E-05** | **3.484E-04** |
| Mean AP | 0.372 | 0.155 | 2.395 | 0.023 | 0.053 |
| **Mean RL** | **0.590** | **0.141** | **4.194** | **1.851E-04** | **6.478E-04** |
| Eccentricity | 0.253 | 0.168 | 1.510 | 0.140 | 0.245 |
| Solidity | -0.153 | 0.171 | -0.894 | 0.378 | 0.378 |
| Orientation | -0.208 | 0.170 | -1.225 | 0.229 | 0.321 |
| Length | -0.160 | 0.163 | -0.983 | 0.333 | 0.378 |
| **C3 metric** |  |  |  |  |  |
| ***Upper*** |  |  |  |  |  |
| **Mean CSA** | **0.577** | **0.143** | **4.033** | **2.94E-04** | **0.001** |
| Mean AP | 0.350 | 0.163 | 2.141 | 0.040 | 0.055 |
| **Mean RL** | **0.635** | **0.136** | **4.683** | **4.41E-05** | **3.08E-04** |
| Eccentricity | 0.365 | 0.162 | 2.249 | 0.031 | 0.054 |
| **Solidity** | **-0.388** | **0.161** | **-2.416** | **0.021** | **0.049** |
| Orientation | 0.013 | 0.174 | 0.074 | 0.941 | 0.941 |
| Length | -0.115 | 0.166 | -0.694 | 0.493 | 0.575 |
| ***Mean*** |  |  |  |  |  |
| **Mean CSA** | **0.555** | **0.145** | **3.836** | **5.17E-04** | **0.002** |
| Mean AP | 0.349 | 0.162 | 2.151 | 0.039 | 0.059 |
| **Mean RL** | **0.614** | **0.137** | **4.466** | **8.37E-05** | **5.86E-04** |
| Eccentricity | 0.344 | 0.163 | 2.115 | 0.042 | 0.059 |
| Solidity | -0.374 | 0.161 | -2.327 | 0.026 | 0.059 |
| Orientation | 0.003 | 0.173 | 0.019 | 0.985 | 0.985 |
| Length | -0.136 | 0.165 | -0.826 | 0.415 | 0.484 |
| ***Lower*** |  |  |  |  |  |
| **Mean CSA** | **0.537** | **0.147** | **3.658** | **8.53E-04** | **0.003** |
| Mean AP | 0.328 | 0.164 | 2.003 | 0.053 | 0.075 |
| **Mean RL** | **0.608** | **0.139** | **4.389** | **1.05E-04** | **7.34E-04** |
| Eccentricity | 0.356 | 0.162 | 2.192 | 0.035 | 0.062 |
| Solidity | -0.373 | 0.161 | -2.322 | 0.027 | 0.062 |
| Orientation | 0.010 | 0.173 | 0.055 | 0.957 | 0.957 |
| Length | -0.152 | 0.164 | -0.925 | 0.362 | 0.423 |
| **SCI-nonNP** |  |  |  |  |  |
| **C2 metric** | **Estimate** | **SE** | **t-value** | **p-value** | **pFDR** |
| ***Upper*** |  |  |  |  |  |
| Mean CSA | 0.271 | 0.238 | 1.138 | 0.271 | 0.780 |
| Mean AP | 0.201 | 0.242 | 0.830 | 0.418 | 0.780 |
| Mean RL | 0.309 | 0.235 | 1.313 | 0.207 | 0.780 |
| Eccentricity | -0.056 | 0.247 | -0.227 | 0.823 | 0.935 |
| Solidity | 0.039 | 0.247 | 0.160 | 0.875 | 0.935 |
| Orientation | 0.020 | 0.247 | 0.082 | 0.935 | 0.935 |
| Length | 0.190 | 0.243 | 0.781 | 0.446 | 0.780 |
| ***Mean*** |  |  |  |  |  |
| Mean CSA | 0.288 | 0.236 | 1.219 | 0.240 | 0.814 |
| Mean AP | 0.231 | 0.240 | 0.963 | 0.349 | 0.814 |
| Mean RL | 0.299 | 0.235 | 1.271 | 0.221 | 0.814 |
| Eccentricity | -0.088 | 0.245 | -0.360 | 0.723 | 0.968 |
| Solidity | 0.010 | 0.246 | 0.041 | 0.968 | 0.968 |
| Orientation | 0.013 | 0.246 | 0.051 | 0.960 | 0.968 |
| Length | 0.135 | 0.244 | 0.553 | 0.587 | 0.968 |
| ***Lower*** |  |  |  |  |  |
| Mean CSA | 0.333 | 0.237 | 1.409 | 0.177 | 0.625 |
| Mean AP | 0.297 | 0.239 | 1.240 | 0.232 | 0.625 |
| Mean RL | 0.276 | 0.241 | 1.146 | 0.268 | 0.625 |
| Eccentricity | -0.168 | 0.247 | -0.681 | 0.505 | 0.884 |
| Solidity | 0.025 | 0.250 | 0.099 | 0.922 | 0.996 |
| Orientation | -0.001 | 0.250 | -0.005 | 0.996 | 0.996 |
| Length | 0.065 | 0.250 | 0.261 | 0.797 | 0.996 |
| **C3 metric** |  |  |  |  |  |
| ***Upper*** |  |  |  |  |  |
| Mean CSA | 0.370 | 0.230 | 1.608 | 0.126 | 0.295 |
| Mean AP | 0.127 | 0.245 | 0.519 | 0.611 | 0.651 |
| Mean RL | 0.524 | 0.212 | 2.474 | 0.024 | 0.170 |
| Eccentricity | 0.462 | 0.220 | 2.097 | 0.051 | 0.180 |
| Solidity | 0.113 | 0.246 | 0.461 | 0.651 | 0.651 |
| Orientation | 0.151 | 0.244 | 0.620 | 0.544 | 0.651 |
| Length | 0.140 | 0.245 | 0.572 | 0.575 | 0.651 |
| ***Mean*** |  |  |  |  |  |
| Mean CSA | 0.392 | 0.227 | 1.728 | 0.102 | 0.238 |
| Mean AP | 0.165 | 0.243 | 0.681 | 0.505 | 0.714 |
| Mean RL | 0.509 | 0.213 | 2.395 | 0.028 | 0.199 |
| Eccentricity | 0.409 | 0.225 | 1.814 | 0.087 | 0.238 |
| Solidity | 0.126 | 0.244 | 0.517 | 0.612 | 0.714 |
| Orientation | 0.149 | 0.243 | 0.611 | 0.549 | 0.714 |
| Length | 0.090 | 0.245 | 0.365 | 0.719 | 0.719 |
| ***Lower*** |  |  |  |  |  |
| Mean CSA | 0.435 | 0.227 | 1.919 | 0.072 | 0.252 |
| Mean AP | 0.230 | 0.244 | 0.946 | 0.357 | 0.626 |
| Mean RL | 0.499 | 0.219 | 2.279 | 0.036 | 0.251 |
| Eccentricity | 0.318 | 0.238 | 1.336 | 0.199 | 0.465 |
| Solidity | 0.168 | 0.247 | 0.681 | 0.505 | 0.691 |
| Orientation | 0.135 | 0.248 | 0.546 | 0.592 | 0.691 |
| Length | 0.027 | 0.250 | 0.109 | 0.914 | 0.914 |
| SCI: spinal cord injury; SE: standard error; FDR: false discovery rate correction; CSA: cross-sectional area; AP: anteroposterior diameter; RL: right-left diameter  Associations surviving FDR correction are in bold | | | | | |
